# Thermal- vs Light-Induced On-Surface Polymerization

**DOI:** 10.1021/acs.jpcc.1c06914

**Published:** 2021-10-08

**Authors:** Christophe Nacci, Monika Schied, Donato Civita, Elena Magnano, Silvia Nappini, Igor Píš, Leonhard Grill

**Affiliations:** † Department of Physical Chemistry, University of Graz, 8010 Graz, Austria; ‡ IOM CNR Laboratorio TASC, 34149 Basovizza, TS, Italy; § Department of Physics, University of Johannesburg, P.O. Box 524, Auckland Park, Johannesburg 2006, South Africa

## Abstract

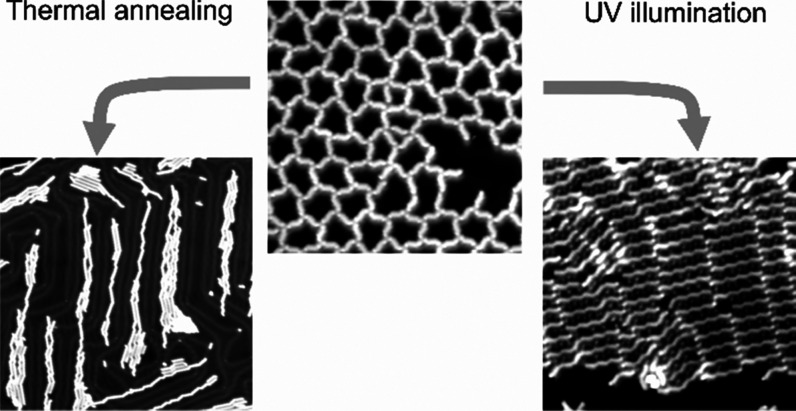

On-surface polymerization is a powerful bottom-up approach that allows for the growth of covalent architectures with defined properties using the two-dimensional confinement of a highly defined single-crystal surface. Thermal heating is the preferred approach to initiate the reaction, often via cleavage of halogen substituents from the molecular building blocks. Light represents an alternative stimulus but has, thus far, only rarely been used. Here, we present a direct comparison of on-surface polymerization of dibromo-anthracene molecules, induced either thermally or by light, and study the differences between the two approaches. Insight is obtained by a combination of scanning tunneling microscopy, locally studying the polymer shape and size, and X-ray photoelectron spectroscopy, which identifies bond formation by averaging over large surface areas. While the polymer length increases slowly with the sample heating temperature, illumination promotes only the formation of short covalent structures, independent of the duration of light exposure. Moreover, irradiation with UV light at different sample temperatures highlights the important role of molecular diffusion across the surface.

## Introduction

On-surface synthesis is a versatile method for the formation of covalently bound nanostructures on surfaces.^1−3^ Ullmann coupling on surfaces is one of the preferred chemical reactions, leading to the formation of C–C bonds.^4−6^ The activation of molecular precursors is operated by dissociating initial C–X (X = I, Br, ...) bonds at room temperature (RT) or at up to about 600 K.^7^ The halogen positions within the precursor define the positions at which new covalent bonds are formed and thus intrinsically encode the final polymer structure.^6,8,9^ Heat, i.e., thermal energy, is the preferred stimulus for activation, but alternative activation modes have also been explored, for instance, a scanning tunneling microscope (STM) tip (via voltage pulses),^10^ an electron beam,^11^ or light.^12^

Light offers numerous advantages with respect to other approaches. The photon energy, polarization, and photon flux can be finely tuned, and this can be reliably done in different environments.^13^ The absence of high temperatures provides better control over precursor diffusion across the surface and circumvents potential problems related to temperature, such as desorption and chemical deterioration of the molecular precursors.

Only a limited number of studies exist on photodriven on-surface chemical reactions compared to processes taking place in gas and solution phases.^13,14^ Though the two-dimensional (2D) confinement of molecular species on the surface decreases the degree of complexity, new challenges are introduced by the role of the molecule–substrate and molecule–molecule interactions.^15^ Accordingly, insight into these fundamental aspects is essential to gain control over light-induced processes on surfaces.

A number of small molecules have been successfully coupled using radiation on HOPG interfaces. Photopolymerization of diacetylene^16^ at the HOPG/liquid interface was triggered by UV irradiation. Methyl-acetylene groups have been activated photochemically on HOPG, resulting in dimer formation via new C–C bonds.^17^ Ordered domains of butadiynyl molecules^18^ were formed at a solid/liquid interface by UV light-induced Glaser coupling reactions. In addition, larger molecules such as C_60_ can be joined by covalent linkage on bulk insulators.^19^ Micrometer-long defect-free polymer fibers were formed on KCl(100) in a chain-growth fashion using UV irradiation.^20^ The C–Br photodissociation of dibromodiantryl precursors on insulating surfaces has been successfully reported.^21^ The photocoupling of fluorinated anthracene triptycene molecules on alkene-passivated graphite surface leads to formation of porous 2D networks ordered on the mesoscale.^22^ Success has also been achieved on metallic surfaces under ultrahigh vacuum (UHV) conditions. The photoisomerization of adsorbed azo-derivative compounds has been reported.^23−27^ The C–Br bond photodissociation of 5,11-dibromo-tetracene molecules adsorbed on Au(111) has been induced by irradiation with visible laser light.^28^ Disordered ensembles of porphyrin derivatives on Ag(110) were converted into squarelike assemblies upon irradiation with visible light for long durations. Furthermore, this leads to the formation of new N=N bonds between porphyrin units.^15^ Photoinduced Glaser coupling of aryl-alkyne molecules on Ag(111) resulted mainly in the formation of dimer species, while the thermal approach led to the formation of long polymers.^29^ Simultaneous C–Br and C–OH bond cleavage of hydroxyphenyl molecules on Ag(111) was triggered by UV light.^12^ This led to the formation of biphenyl biradical species that assemble into porous networks, stabilized by interactions with substrate Ag atoms at 80 K. At RT, the same radical species couple to each other and form covalently linked polyphenylene chains.

Chemical bonds within adsorbed molecules have been dissociated using either the STM tip,^10,30^ heat,^2^ or light^12^ or by a combination of voltage pulses from a STM tip and light.^31,32^ The subsequent synthesis of covalently linked nanostructures is achieved by thermal activation^4^ or without heating (i.e., at RT) in the case of photoactivated species.^12^ The crucial role of species diffusion has been highlighted by exploring photodriven Glaser coupling reactions on different (111) metal surfaces.^29^ However, a systematic comparative investigation of the heat- and light-driven processes from intact molecules to polymers is missing so far. It is of interest as to whether these processes evolve similarly or deviate from each other and lead to equivalent or different intermediate molecular assemblies and covalent structures. In this sense, it is important to identify in both approaches (thermally- and light-induced) the processes at work, how they compete with each other, and the role each plays in on-surface synthesis. Here, we report a comparison of thermally and photoinduced on-surface polymerization of the same molecular precursor on a Au(111) surface using low-temperature scanning tunneling microscopy and high-resolution core-level X-ray photoemission spectroscopy (XPS). Thermally and light-triggered homocoupling reactions result in the formation of polymers, but their growth evolves differently. Long polymers are achieved by increasing the sample annealing temperature, while UV irradiation results in shorter oligomers. This is independent of the illumination time, i.e., the amount of delivered energy, because the process is diffusion-limited.

## Methods

### Sample Preparation

The Au(111) sample was cleaned by repeated argon ion sputtering (1.3 keV) and subsequent annealing at 770 K. DBA molecules were deposited from a Knudsen cell (sublimation temperature of 388 K) onto Au(111) held at room temperature.

### Scanning Tunneling Microscopy Measurements

Scanning tunneling microscopy images were recorded in constant-current mode at 5 and 7.5 K with the bias voltage referring to the sample with respect to the grounded STM tip.

### Sample Irradiation

Samples were illuminated in UHV conditions using a CW solid-state laser (CryLas) with a nominal wavelength of 266 nm (power of 6.10 mW). The laser beam spot hitting the sample surface was approximately circular with a diameter of about 4.0 mm. The Au(111) crystal sample has an area of 7 × 7 mm^2^. The laser light entered the UHV preparation chamber via a MgF_2_-coated UHV viewport that ensured a transmission coefficient of about 90% in the 200–6000 nm wavelength range. Samples were about 36.0 cm away from the laser source, and the illuminations were carried out in normal incidence conditions. The sample temperature remained stable during the illumination process.

### XPS Measurements

High-resolution XPS spectra were obtained at the BACH^33,34^ beamline at the ELETTRA synchrotron (Trieste, Italy) in an UHV system comprising a preparation chamber and an analysis chamber equipped with a hemispherical electron-energy analyzer (Scienta R3000, VG Scienta).^35^ A photon energy of 380 eV and a total instrumental resolution of 210 meV were employed to excite the C 1s and Br 3d core levels. All spectra were acquired at an emission angle of 60° from the surface normal. All binding energies are stated relative to the Fermi level. The C 1s spectra were deconvoluted into Voigt line shapes, and the Br 3d spectra were fitted using Voigt doublet line shapes. The temperature-dependent C 1s and Br 3d XPS spectra were continuously acquired by ramping the sample temperature from RT up to 280 °C. The heating rate during the temperature-programmed XPS measurements was 12 °C/min. Both C 1s and Br 3d spectra were recorded during the temperature ramp with a 5.6 °C temperature difference between two successive carbon or bromine spectra.

## Results and Discussion

**Figure 1:**
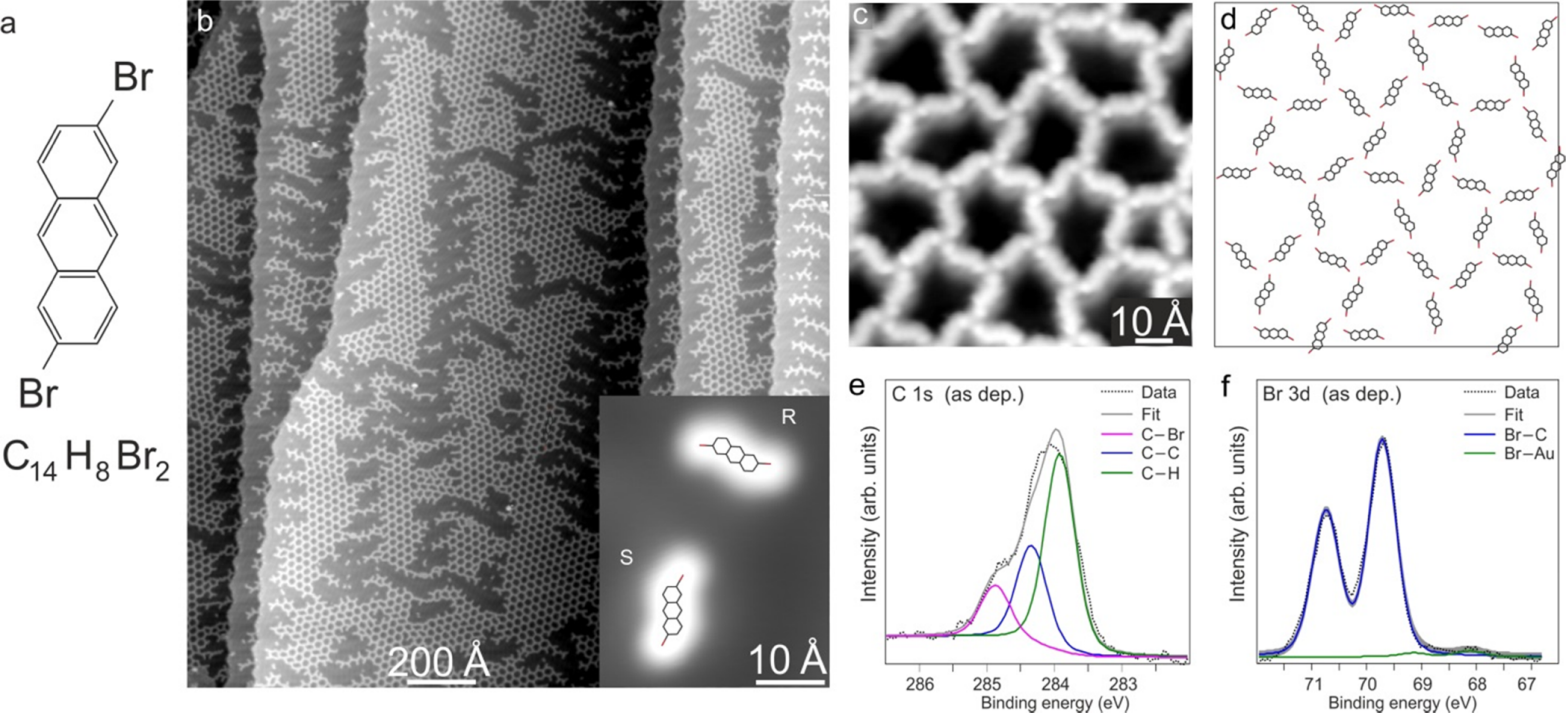
Adsorption of molecular precursors. (a) Chemical structure of the DBA molecule. (b) Scanning tunneling microscopy overview image (0.5 V, 55 pA) of 0.5 ML DBA on Au(111), showing intact molecules in extended assemblies. The inset shows individual intact DBA molecules (0.2 V, 10 pA). Both enantiomers R and S are observed (as indicated). The chemical structure is superimposed on both of them. (c) Scanning tunneling microscopy image of a porous network with a functionalized STM tip (50 mV, 4 pA). (d) Sketch of the network structure shown in (c). (e) C 1s and (f) Br 3d XPS spectra (hν = 380 eV) taken on 0.5 ML DBA/Au(111) at RT. All scanning tunneling microscopy images were taken at 7.5 K.

The molecule under investigation is 2,6-dibromo-anthracene (C_14_H_8_Br_2_, DBA henceforth). As this anthracene-derivative precursor carries two Br atoms in trans configuration (Figure 1a), covalently bound chains are expected to be formed on the surface. These are convenient model structures to explore the on-surface reaction details using scanning tunneling microscopy-based structural characterization. Molecules were deposited onto Au(111) kept at RT, and the scanning tunneling microscopy measurements were conducted at 5 and 7.5 K and XPS measurements at RT. DBA molecules adsorb intact on the surface and self-assemble into two-dimensional porous networks and open structures (Figures 1b and S3a). Scanning tunneling microscopy imaging of individual DBA molecules (inset of Figure 1b) reveals a clear structural asymmetry related to the two C–Br bonds pointing in different directions, as shown by the model structures superimposed on the image. Consequently, the adsorbed molecules are chiral and both enantiomers are found on the surface (inset of Figure 1b). Individual DBA molecules were also clearly resolved within self-assembled porous structures (Figure 1c): the internal structure of these networks is based on nodes of neighboring C–Br bonds (see the model in Figure 1d). The local halogen interactions provide a suitable balance between van der Waals and electrostatic interactions^36^ that favors the formation of extended porous networks.

The chemical state of the molecular species was analyzed using high-resolution C 1s and Br 3d XPS spectra. The C 1s spectrum of 0.5 monolayer (ML) DBA/Au(111) at RT (Figure 1e) reveals two clear features: a dominant one centered at about 284.1 eV and a minor one (purple) at higher binding energies (BE) (284.9 eV). The former is ascribed to the adsorbed anthracene units.^28,37^ The asymmetric shape of this peak is modeled with two components at 283.9 and 284.3 eV (green and blue, respectively) attributed to hydrogenated (C–H) and nonhydrogenated (C–C) C atoms, respectively.

**Figure 2:**
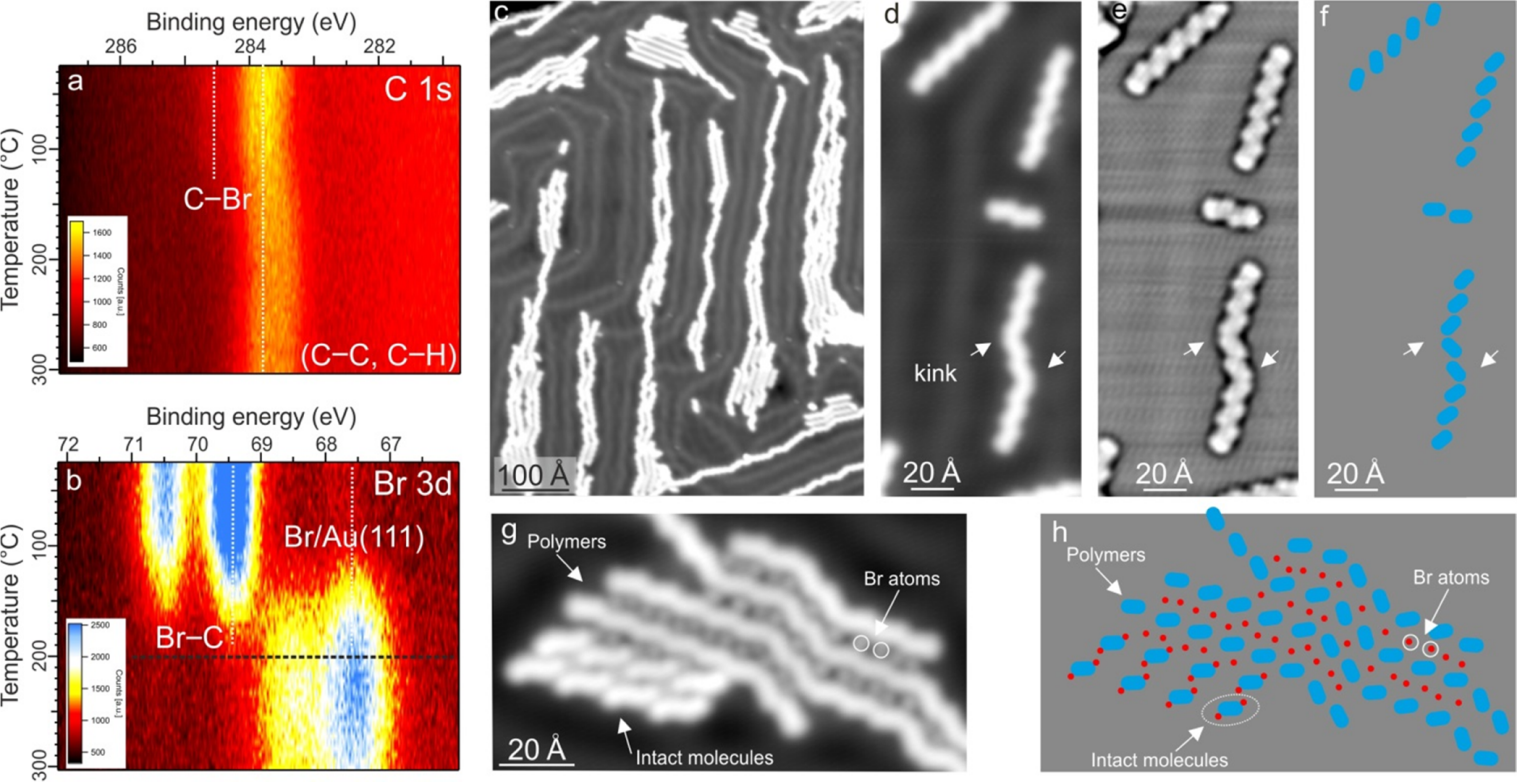
Thermally induced on-surface polymerization. 2D intensity maps show the temperature dependence (obtained at a rate of 12 °C/min) of (a) C 1s and (b) Br 3d (hν = 380 eV) XPS spectra taken on 0.5 ML DBA/Au(111). (c) Large scanning tunneling microscopy overview of thermally grown polymers (0.1 V, 100 pA), (d) individual oligomers (0.1 V, 80 pA), and (e) Laplace-filtered image enhancing the internal structure of the oligomers in (d). (f) Sketch of the oligomer internal structure in (d, e). (g) 2D islands of close-packed polymers (0.1 V, 80 pA); neighboring polymers are separated by Br atoms. Close-packed intact molecular precursors are identified too. (h) Sketch of the structures in (g). Blue ellipses and red circles represent anthracene units and Br atoms, respectively. All covalent structures in the figure were obtained by depositing DBA molecules on the Au surface kept at RT. The covalent structures were obtained by ramping the sample temperature from RT up to 230 °C. All scanning tunneling microscopy images were taken at 7.5 K.

The component at 284.9 eV is attributed to the C atoms linked to the Br atoms (C–Br).^18,37−39^ On the other hand, the Br 3d spectrum (Figure 1f) is dominated by a Br 3d doublet with Br 3d_5/2_ centered at 69.7 eV (blue) and attributed to the Br–C bonds.^38^ A minor Br 3d doublet with Br 3d_5/2_ (green) shifted about 1.6 eV toward low BE is required to achieve better peak deconvolution (see also Figure 2b) and ascribed to surface-bound Br atoms.^40,41^ The latter corresponds to about 3% of the entire Br 3d signal. Accordingly, almost all adsorbed molecules are intact, which is expected due to the low Au(111) surface reactivity. If the surface is heated to different temperatures after molecule deposition, the C 1s and Br 3d core levels change in a characteristic manner (Figures 2a,b and S1): a continuous depletion of the C–Br component is observed, which we ascribe to the progressive debromination of the adsorbed DBA. At the same time, ongoing on-surface polymerization is evidenced by the increase of the C–C component due to the formation of new C–C covalent bonds (Figure S1a). Debromination is completed at about 200 °C at the used temperature ramp (see Methods) as suggested by complete depletion of the Br 3d spectroscopic feature attributed to the Br–C bond at the high BE side (Figures 2b and S1b). The separated Br atoms are also visible in the scanning tunneling microscopy images (Figure 2g). Moreover, the C 1s peak is shifted by about 0.30 eV toward low BE in the explored range of temperature. Importantly, this affects all components of the C 1s feature (Figure S2a). Such a shift has been observed before and was ascribed to the Au surface work function increase, due to surface chemisorbed Br atoms.^37,38,42^

Note that after heating to at least 100 °C, two doublet components are needed to describe the Br 3d spectroscopic feature at low BE (Figure S1b). They may be ascribed to Br atoms adsorbed on different sites,^40^ for instance, terraces and step edges or different adsorption sites on the herringbone reconstruction. Further annealing up to 280 °C left all core levels unchanged, which is a signature of the high chemical stability of the on-surface grown polymers and chemisorbed Br atoms. Note that the total Br 3d signal intensity decreases with temperature (Figure S2b), due to a thermally induced Br loss (see Supporting Information, Figure S2).

When following the same process of thermally induced on-surface polymerization by scanning tunneling microscopy, covalently linked linear structures are observed in both isolated and close-packed configurations (Figure 2c). These polymers consist of straight segments and kinks (Figure 2d). The anthracene constituent units are regularly organized along the polymer axis as highlighted by the Laplace-filtered scanning tunneling microscopy images (see Figure 2e and the sketch in Figure 2f). If the debromination process is not completed, 2D close-packed islands of intact molecules are formed (Figure 2g and the sketch in Figure 2h). After sample heating, porous networks are no longer observed, which indicates that the close-packed islands are thermodynamically more stable than the porous networks.

**Figure 3:**
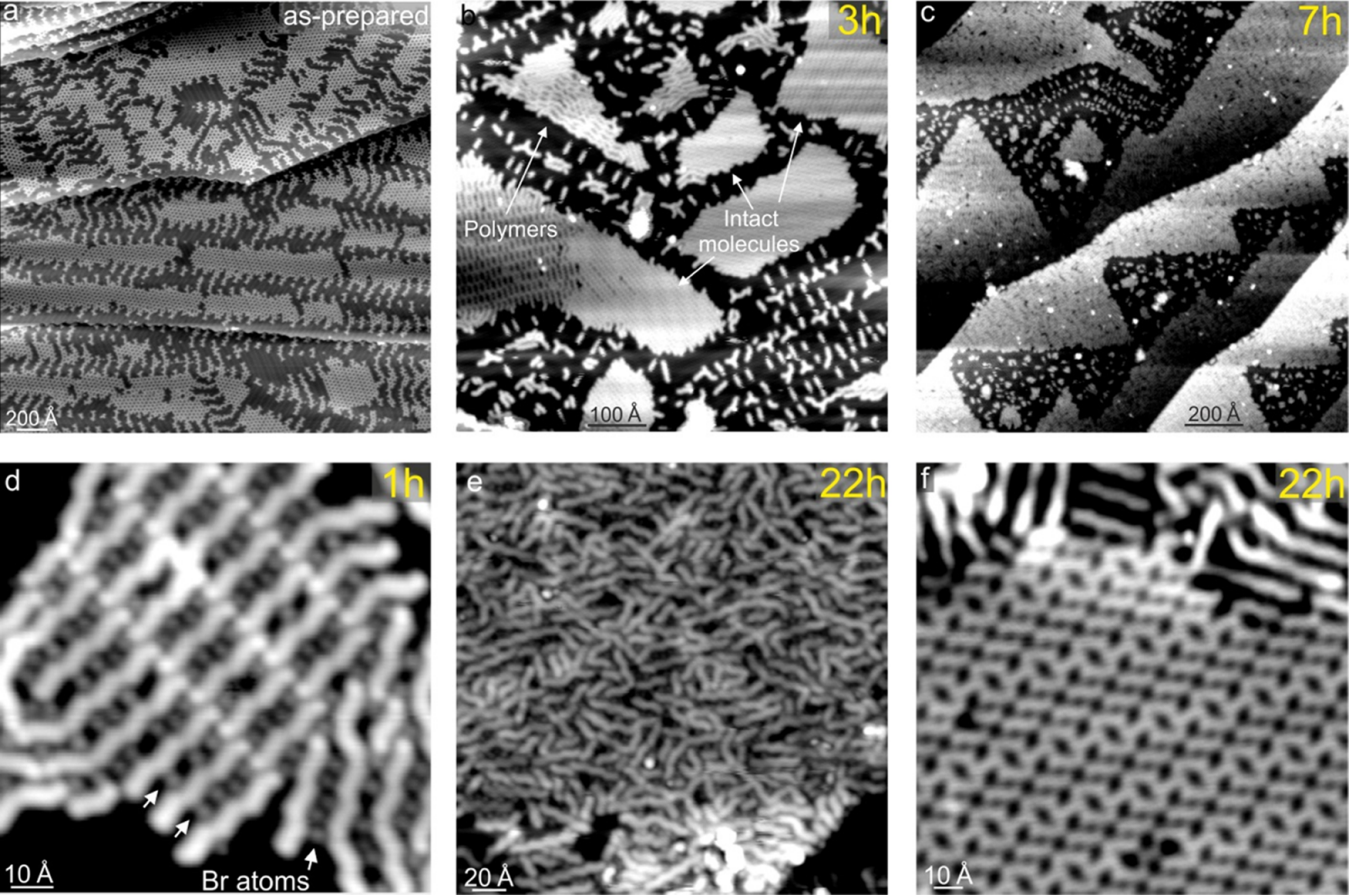
Illumination with UV light. Scanning tunneling microscopy images of 0.5 ML DBA/Au(111) taken at 7.5 K: as-prepared (a) and after UV (266 nm) laser light illumination at room temperature (3 h (b) and 7 h (c)). Image (c) shows a large disordered island of oligomers, but ordered islands are still present after 7 h of UV illumination. (d) 2D close-packed oligomers after 1 h of illumination. (e) Ensemble of disordered polymers. Neighboring polymers are separated by Br atoms as indicated in (d). (f) Zoom-in of a 2D close-packed arrangement of intact DBA molecules. Images (e, f) were obtained after 22 h of illumination.

In addition to thermally induced polymerization, we also tested the photoinduced polymerization: 0.5 ML DBA/Au(111) was illuminated with UV (266 nm) light at RT and afterward characterized using scanning tunneling microscopy. After irradiation, the original porous networks (Figures 1b,c and 3a) become partially converted into 2D close-packed arrangements. These are made of either intact molecules (Figure 3b) or oligomers (Figure 3d,e), which is a signature of successful photoinduced C–Br dissociation and chemical coupling of the molecular precursors. Close-packed oligomers are typically separated from each other by rows of Br atoms (Figure 3d). Importantly, both thermal- and photoassisted growth approaches lead to the formation of structurally equivalent oligomers (Figures S4 and S5), which rules out possible light-induced side effects such as molecular damage.^43^ Regarding the precise adsorption geometry, the in-plane orientation of the anthracene axis with respect to the oligomer axis/orientation of the close-packed oligomers is a factor of 2 smaller than that of the isolated oligomers (Table S1). This suggests that the internal oligomer structure within close-packed structures is strongly affected by the local environment, and the significant “straightening” of the anthracene units can tentatively be ascribed to an oligomer–halogen interaction^44^ (see Figures S4 and S5).

**Figure 4:**
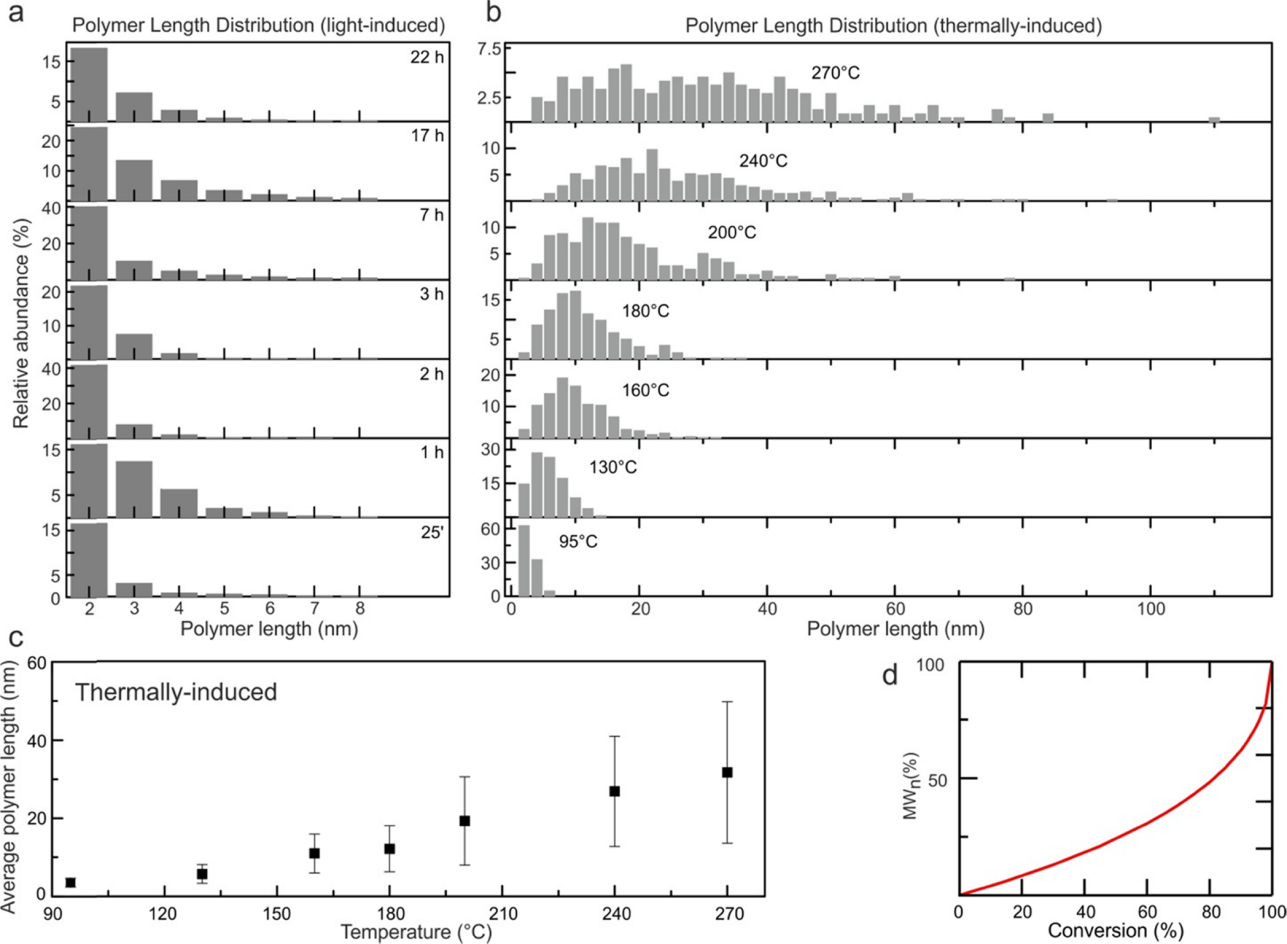
Polymer length distribution. (a) Polymer length distribution after UV (266 nm) laser light illumination. (b) Polymer length distribution as a function of the sample annealing temperature. The relative abundance of monomers is not included in both histograms for the sake of clarity (see Figure S6). Note that each monomer has a length of about 1 nm (i.e., a 10 nm long polymer is composed of 10 monomer units). (c) Average polymer length as a function of the sample annealing temperature. The error is the standard deviation of the corresponding data set. (d) Schematic step-growth polymerization curve (normalized molecular weight MW_n_ vs conversion), according to Flory’s model.^46^

To understand how UV illumination affects the oligomer growth, a large range of illumination times was probed, from few tens of minutes up to 22 h (Figures 3 and 4).

Qualitatively, all oligomer length distributions follow the same behavior over the entire illumination range (Figure 4a). Apparently, the illumination time is not crucial for the final product distribution as extended UV illuminations do not change the relative abundances of the on-surface oligomer products. Intact monomers (Figure 3f) typically represent the most abundant species (Figure S6), and oligomers longer than four anthracene units are a small minority (about a few % of all species, see Figure S7). The growth of the latter is disfavored in comparison to the shortest oligomers even after extended UV irradiation. In all cases, irradiation was conducted with the sample kept at RT. Accordingly, thermal diffusion across the surface is similar in all irradiation experiments. This points out that the sample temperature plays a relevant role. On the other hand, substantial differences are observed when inducing polymerization thermally, i.e., by heating the sample to various temperatures (Figure 4b) as the maximum of each length distribution shifts to higher values, as does the average polymer length (Figure 4c), which is in contrast to the light-induced process. In addition, the distributions get broader with increasing temperature. This is because annealing at higher temperatures has two effects: it enables completion of the debromination process (see XPS spectra in Figure S1) and enhances diffusion of the debrominated species across the surface, promoting the growth of new polymers and elongation of the existing ones. Consequently, the average polymer length is higher for thermal polymerization than for the photoactivated approach (Figure 4).

It is well known from solution chemistry that polymerization can occur via step-growth^1^ or chain-growth.^20^ The former is characterized by an initially slow increase of the average polymer length and large polymers only appear at very high rates (Figure 4d), while the latter exhibits a much steeper increase from the very beginning.^45^ Our experiments show a rather slow and approximately linear increase of the average polymer length with temperature (Figure 4c). This is in good agreement with the predictions for the early–middle stages of a step-growth model as shown in Figure 4d. The prevalence of rather low-molecular-weight covalent structures and a broad distribution of molecular weights are typical aspects for step-growth^1^ as long as the conversion is not completed, i.e., the conversion does not reach very high values^45^ (Figure 4d). Note that a conversion of only about 60% was determined in our experiments, even for the very high temperature of 270 °C. Here, the molecules are confined on a surface and the extent of diffusion is reduced if the oligomers become longer, due to the increasing adsorption energy. This agrees with our observation of a slowly increasing polymer length with the temperature in combination with a relatively broad distribution (Figure 4b,c).

**Figure 5:**
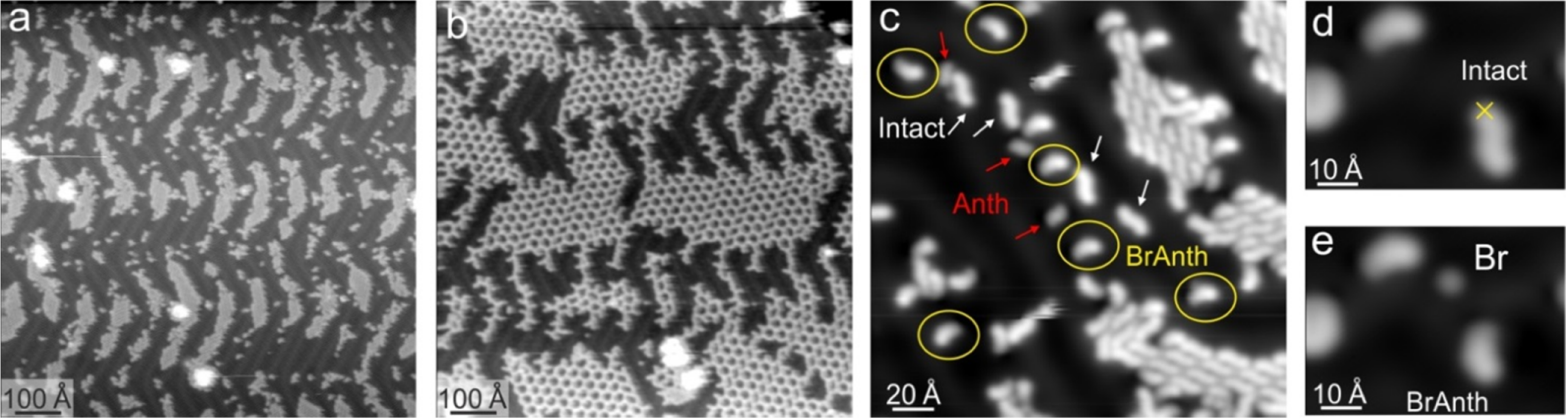
UV illumination of 0.5 ML DBA/Au(111) at 77 K. (a) Large scanning tunneling microscopy overview image taken in the proximity of the laser beam spot’s center after 6 h of continuous illumination. (b) Large scanning tunneling microscopy overview of a nonilluminated area showing intact molecules arranged into porous networks. (c) A zoom-in of (a) shows 2D islands made of intact molecules surrounded by either individual intact or photo-debrominated species. (d) Voltage pulse-induced debromination of an individual intact molecule. The STM tip is placed at the cross position and the bias voltage ramped up to 2.2 V. (e) Same area of (d) after pulsing. The reaction products are labeled accordingly. The scanning tunneling microscopy images were acquired at 5 K.

To further explore the role of temperature and to understand how light affects the various molecules (precursors and products) with reduced thermal diffusion on the surface, we performed UV (266 nm) illumination keeping the sample at 77 K. After 6 h of continuous illumination, 2D islands of mainly intact molecules are found (Figure 5a). Porous networks are only found in nonilluminated areas (Figure 5b). Partially debrominated species lose the typical asymmetry of intact molecules (inset of Figure 1b) and appear curved (the molecule’s dim termination is the position at which the Br atom is missing; Figure 5c). Voltage pulses applied to intact molecules lead also to the formation of partially (Figures 5d,e and S8a) and fully debrominated precursors (Figure S8b), which are identical in appearance to the photoinduced ones. Note that after irradiation at 77 K, fewer Br atoms are observed near the molecules than for illumination at room temperature. This could mean that Br atoms adsorb at step edges while in the case of RT preparation, the Br atoms are trapped between the oligomers (Figure 3d). Photodriven debromination thus takes place on the sample kept at 77 K and with the same efficiency as at RT (see Supporting Information, page S11). However, the lack of thermal energy suppresses the mobility of the activated species such that polymer growth is completely inhibited. Individual intact molecules can instead still diffuse at 77 K and form porous networks (Figure S9). This highlights the crucial importance of the temperature within the photodriven polymer growth scheme.

## Conclusions

In summary, this work shows that debromination and on-surface polymerization of the same molecular precursors can be triggered by either thermal annealing or UV illumination. Both approaches are successful in driving C–Br bond dissociation and formation of covalently linked linear structures. Nevertheless, our structural characterization reveals subtle differences between the thermal- and the light-activated approaches.

Increasing UV exposure to the surface, kept at room temperature, does not induce substantial changes in terms of the relative species abundance and polymer length distribution, indicating diffusion-limited polymer growth. On the other hand, thermal activation of molecular precursors allows for better tuning of the species mobility. In the regime of mild thermal annealing, short oligomers comprising few units are found, similar to the photoassisted case. Long polymers grow only at high temperatures, owing to the enhanced Br dissociation and molecular mobility. If the surface temperature is lowered to 77 K during light exposure, polymer growth is totally suppressed because the activated species can no longer diffuse while the intact molecules are still mobile.

## Supporting Information Available

C 1s and Br 3d temperature-dependent XPS spectra; binding energy shift and core-level peak areas vs sample annealing temperature; 2D close-packed arrangements of intact molecules and oligomers; internal structure of the oligomers; structural characterization of the oligomers; polymer length distributions (light- and thermally induced); polymer distribution length (photoapproach); debromination by voltage pulses; photo-debromination efficiency at room temperature and 77 K; and porous networks formation at 77 K (PDF)
