# Supplementary material for: Thermal- vs Light-Induced On-Surface Polymerization
Source: J Phys Chem C Nanomater Interfaces. 2021 Oct 8;125(41):22554–61. doi: 10.1021/acs.jpcc.1c06914 (PMC8543439; doi:10.1021/acs.jpcc.1c06914)
Supplement: Supplementary file 1 — jp1c06914_si_001.pdf [file jp1c06914_si_001.pdf]

Thermal- vs Light-Induced On-Surface Polymerization

*Christophe Nacci**[,1], Monika Schied[1], Donato Civita[1], Elena Magnano[2,3], Silvia Nappini[2], Igor Píš [2], and Leonhard Grill**[,1]

1) Department of Physical Chemistry, University of Graz, 8010 Graz, Austria

2) IOM CNR Laboratorio TASC, 34149 Basovizza (TS), Italy

3) Department of Physics, University of Johannesburg, P.O. Box 524, Auckland Park, Johannesburg 2006, South Africa

* Email: christophe.nacci@uni-graz.at

* Email: leonhard.grill@uni-graz.at

Supporting Information

Table of contents

1) C 1s and Br 3d temperature-dependent XPS spectra S2
2) Binding energy shift and core level peak areas vs sample annealing temperature S3
3) 2D close-packed arrangements of intact molecules and oligomers S5
4) Internal structure of the oligomers S6
5) Structural characterization of the oligomers S7
6) Polymer length distributions (light- and thermally-induced) S9
7) Polymer distribution length (photoapproach) S10
8) Debromination by voltage pulses S11
9) Photo-debromination efficiency at room temperature and 77 K S11
10) Porous networks formation at 77 K S12

1. C 1s and Br 3d temperature-dependent XPS spectra

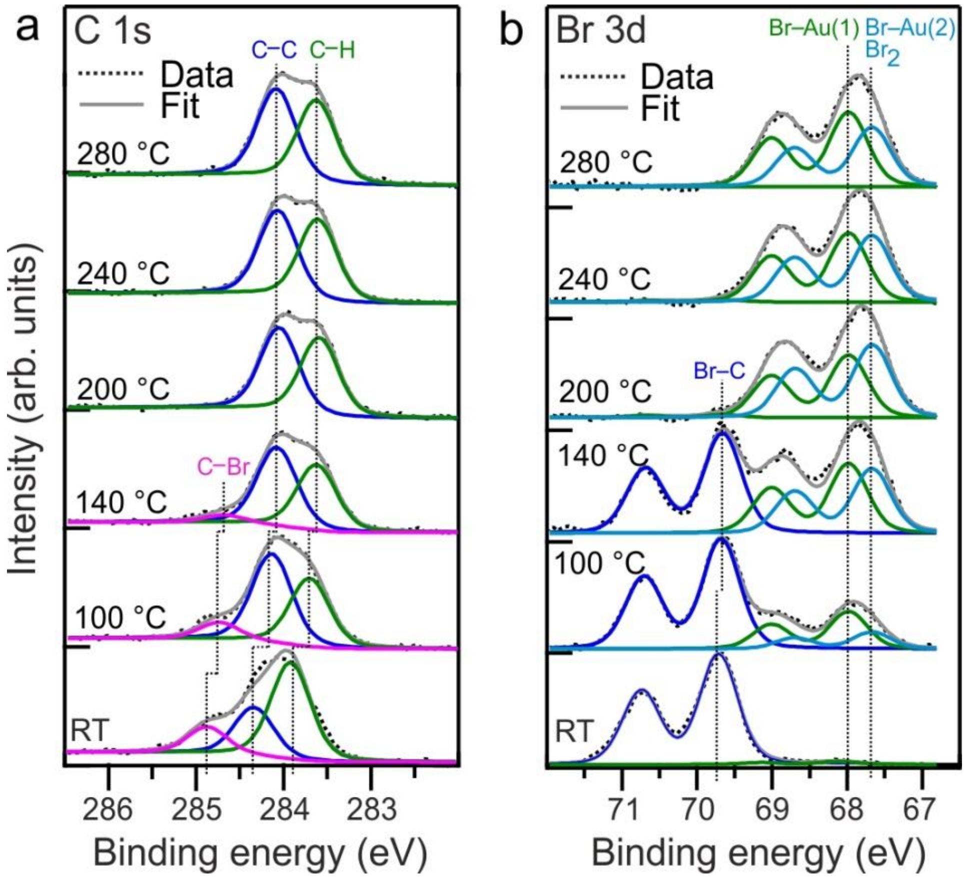

Figure S1. (a) C 1s and (b) Br 3d temperature-dependent XPS spectra (hν= 380 eV). XPS measurements were taken at room temperature after the sample thermal annealing. The temperature values close to each spectrum indicate the sample heating temperature.

High-resolution C 1s and Br 3d core level XPS spectra (hν= 380 eV) were acquired at room temperature (RT) after thermal annealing (see temperature values indicated in panels (a,b)). The C 1s core level has been deconvoluted with three (Voigt function) components (C–Br, C–C and C–H), the intensity ratios C–H/C–C (1.99) and C–H/C–Br (3.69) are in good agreement with the stoichiometry of the intact molecules (C–H : C–C : C–Br = 8 : 4 : 2). The ongoing debromination process is evident from the progressive depletion of the C-Br component at high BE. The increase of the C–C components is ascribed to the formation of new C–C covalent bonds. Accordingly, the C-Br component of the Br 3d core level is also depleted at about 200 °C (Fig.2b in the main text). The C–H/C–C ratio at 280 °C is 0.93 while the expected ideal ratio is 1.33 (C–H : C–C = 8 : 6).

After annealing, two components are needed to fit the Br 3d feature at low binding energies (corresponding to Br that is not bound to the molecules). They have been assigned in previous studies either to two chemically different Br atoms[1] (for instance adsorbed on terraces and step edges) or to surface-bound Br and Br_2, respectively[2].

2. Binding energy shift and core level peak areas vs sample annealing temperature

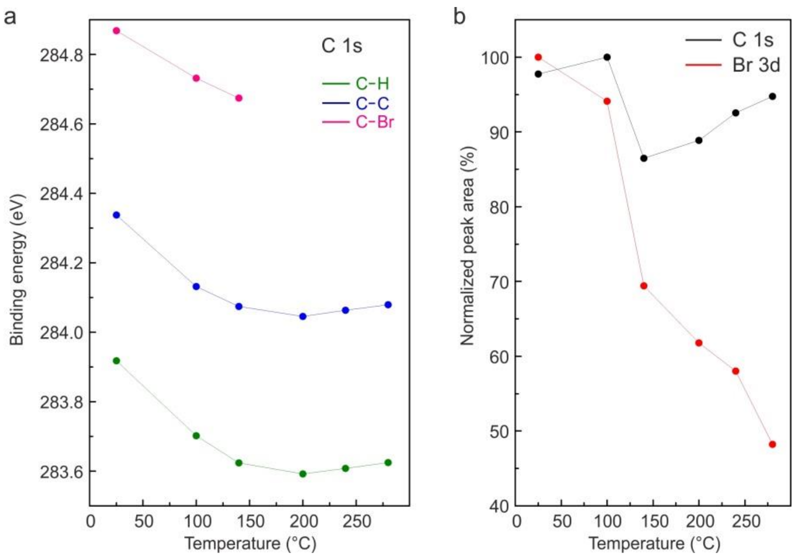

Figure S2. (a) BE shift of all C 1s core level components (C─C, C─H and C─Br) as a function of the sample annealing temperature. (b) Normalized peak areas of C 1s and Br 3d core levels spectra as a function of the sample annealing temperature. The peak area is normalized to its maximum value.

The C-C and C-H components of C 1s experience an overall shift of about 300 meV to lower binding energy (BE) as a function of the sample annealing temperature. The C-Br component is shifted by about 200 meV and disappears at 200°C (Figures S1a,b). The C-C and C-H components both reach a plateau at about 200 °C (Fig.S1a) where the debromination process is approximately completed (Figures 2a,b and Fig.S1).

Such a shift can be ascribed to the energy level alignment at the organic-metal interface[3,4]. In weakly interacting adsorbate-substrate systems, changes in the vacuum level rigidly shift all adsorbate levels by the same amount. Br atoms spilled over the gold substrate upon dissociation are increasing the substrate work function, which correlates with the C 1s shift to lower energies[5–7].

The Br 3d intensity drops significantly with the temperature while the C1s intensity remains roughly constant (Fig.S2b). The Br loss might be ascribed to the desorption of atomic Br from the surface[8]. Furthermore, Br atoms might combine with atomic H and desorb as HBr[9–12]. Similarly, Br atoms might bind to each other and desorb as Br_2[8].

The desorption of Br atoms from the Au(111) surface takes place by thermal annealing beyond 523 K[8,9,13]. The intact brominated species self-assemble into porous networks when deposited on Au(111) without heating (i.e., at RT) (see Fig.1 in main text). Thermally-induced close-packed structures are observed (Fig.S3). Accordingly, thermal annealing induces the adsorption of Br atoms likely close to each other. This likely favors the formation and possibly their desorption as Br_2 molecules[14].

Removing halogen byproducts from a surface by exposure to a beam of atomic hydrogen is a common methodology[9–11]. In this regard, exposures in a background pressure of 10^{-7} mbar H_2 are used while keeping the substrate at about 500 K. The H_2 background in the UHV chamber where our samples were prepared and measured is about 2-3 order of magnitude better than the value needed to efficiently remove Br atoms from the surface. The atomic H might also originate from the C-H cleavage of the adsorbed species. The onset of the dehydrogenation processes begins at around 550 K[9]. Accordingly, the observed Br loss as a function of the temperature (Fig.S2b) might take place mainly through the formation of Br_2[8].

3. 2D close-packed arrangements of intact molecules and oligomers

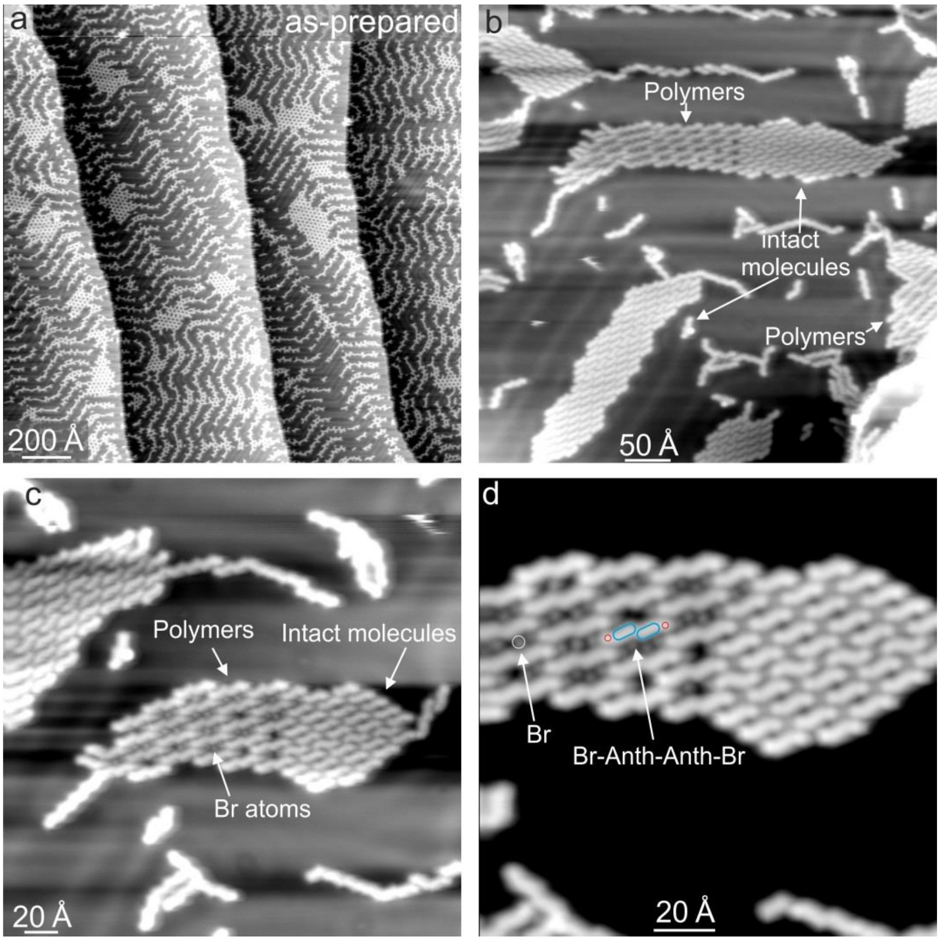

Figure S3. (a) Large STM overview of DBA/Au(111). Molecules are mostly arranged into porous networks and open structures. (b,c) 2D close-packed islands made either of intact molecules or short oligomers. (d) Zoom-in of panel (c). Individual Br atoms and Br-terminated dimers are indicated. The structures observed in (b-d) were obtained by thermal annealing from 45°C up to 95 °C in 14 minutes. Afterward the sample was cooled down to RT in about 10 minutes and then transferred into the cold STM. All STM images were taken at 5 K.

After depositing molecules onto the Au substrate, kept at room temperature, the molecules are arranged in extended porous networks and open structures (Fig.S3a). Close-packed islands comprised either of intact molecules or short oligomers (Figures S3b,c) are found after mild sample annealing (95 °C in this case). Oligomers are separated from each other by individual round features that we assign to Br atoms as they were missing before the sample annealing. Individual dimers appear still terminated with Br atoms (Fig.S3d) because of the partial debromination obtained at mild conditions (see also Figures S4 and S5 and related discussions).

We rule out that oligomers are Au-terminated as we do not see a C-Au component in the C 1s XPS spectra. Moreover, the number of round features identified between oligomers and the ones at the polymer termini can be compared to the expected number of Br atoms provided by the debromination of the anthracene units within the same cluster. A ratio equal or greater than 60% is found between the number of identified round features and the expected Br atoms. Accordingly, we assign those round features to Br atoms.

4. Internal structure of the oligomers

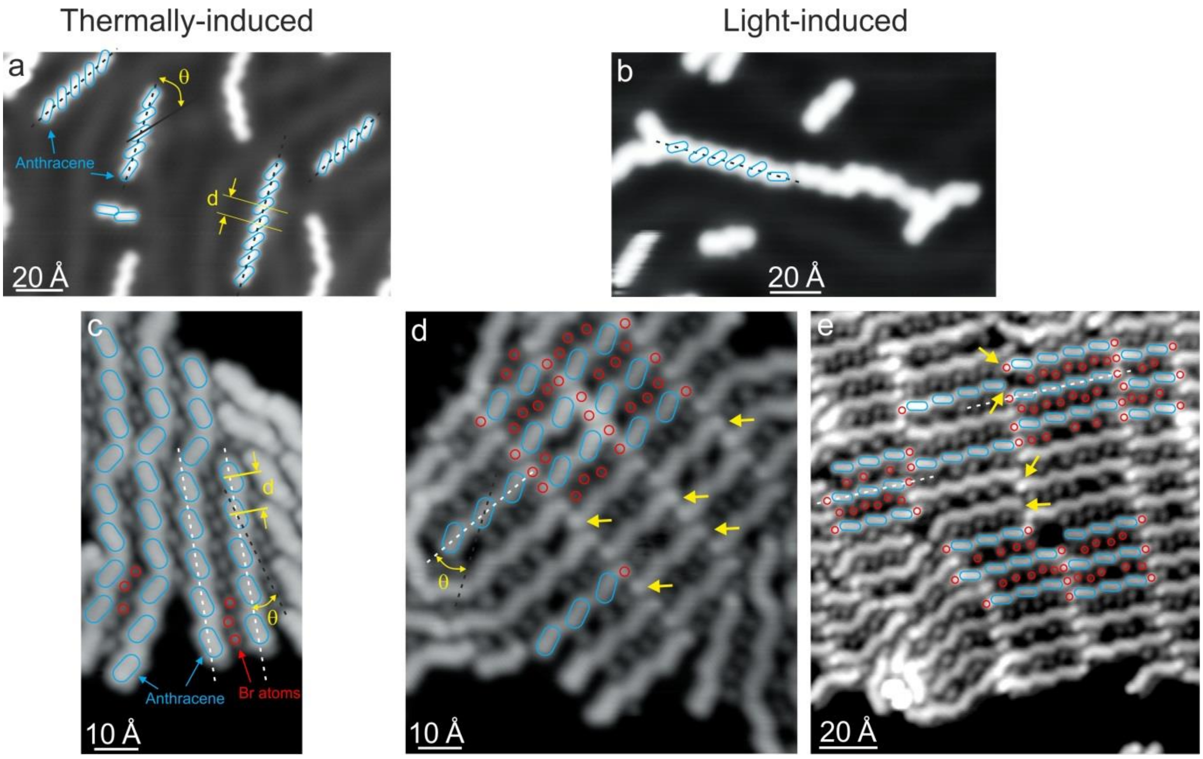

Figure S4. Isolated (a) and close-packed (c) oligomers obtained by thermal annealing up to 250 °C. Isolated (b) and close-packed (d,e) oligomers obtained by 1 h of UV (266 nm) laser illumination. A model sketch is superimposed to the STM images in order to highlight the internal structure of the oligomers. The anthracene units are indicated by blue empty ellipses while the Br atoms are indicated by red empty circles. The oligomer axis is indicated by a dashed line. The angle ϑ between the anthracene units and the oligomer axis and the distance *d* between neighboring anthracene units are shown.

Oligomers are found on the surface as isolated entities (Figures S4a,b) as well as organized in close-packed islands (c-e). In the latter case, oligomers are separated from each other by Br atoms supplied by the debromination processes (either thermally or light-induced). In some cases, the oligomer termini reveal a clear asymmetric shape - see arrows in Figures S4d,e. Oligomers are indeed in some cases still terminated with Br atoms as a signature of a not completed debromination process (Figures S4d,e). The round features/expected Br atoms ratio (see Br counting argument proposed in section 3 above) is about 97% and 93%, obtained from STM images (Figures S4d and e, respectively). The same ratio results significantly lower in the case of thermally induced debromination (about 60% calculated from Fig.S3), likely because of the Br desorption.

A systematic comparison of anthracene wires obtained either by thermal or photo-approach was done by assessing structural parameters as the angle ϑ between individual anthracene units and the polymer axis (see Figures S4a,c), the distance *d* between neighboring anthracene units within the same polymer (Fig.S4a) and the polymer apparent height. See next section (S5) for more details.

5. Structural characterization of the oligomers

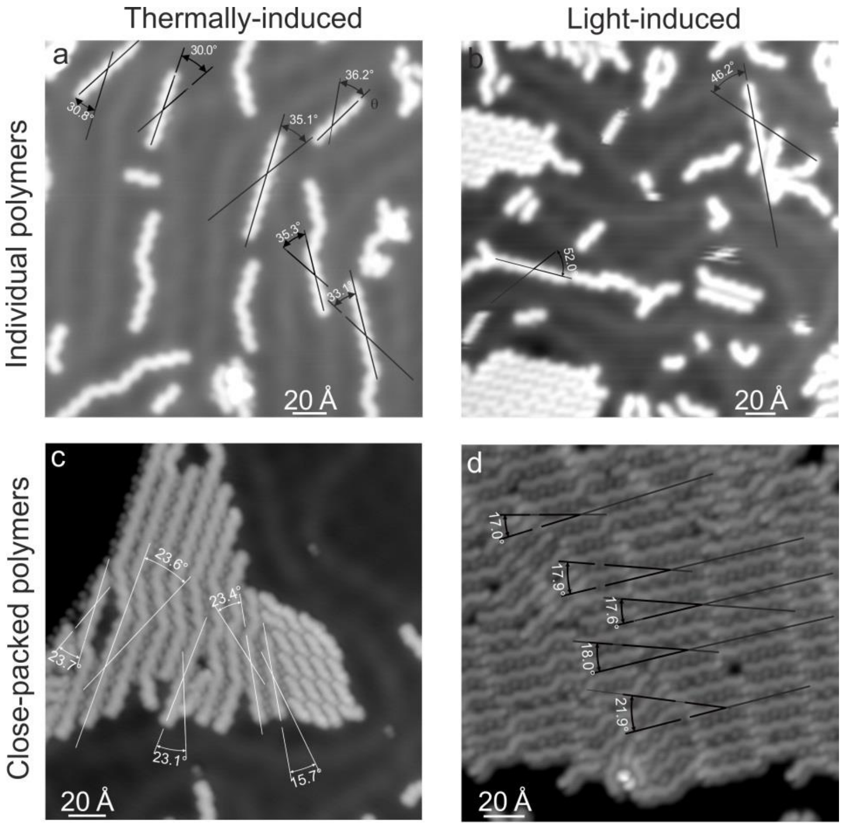

Figure S5. Representative STM images of individual (a) and close-packed oligomers (c) obtained by thermal annealing up to 250°C. Individual (b) and close-packed (d) oligomers after 1 h of UV (266 nm) illumination.

	Approach	ϑ(°)	Distance d (Å)	Apparent height (Å)
Individual oligomers	Thermo	39.6 ± 7.1	9.5 ± 0.5	1.59 ± 0.06
	Photo	44.7 ± 8.0	9.8 ± 0.3	1.57 ± 0.04
Close-packed oligomers	Thermo	24.0 ± 4.5	8.8 ± 0.4	1.49 ± 0.11
	Photo	20.1 ± 3.0	9.5 ± 0.7	1.51 ± 0.13

Table S1. STM structural characterization of oligomers found either isolated or close-packed into islands obtained by using the thermo- and photo-approach. The angle θ between the anthracene units and the polymer axis, the distance d between neighboring anthracene units within the same polymer and the polymer apparent height are reported. The characterization is made on a statistical basis. Each value represents the average value, the error is the standard deviation σ value of the corresponding distribution. Regarding the individual oligomers, 18 wires (thermo) and 3 wires (photo) have been evaluated. Concerning the 2D oligomer islands, 11 wires have been measured for each approach.

An STM-based characterization of polymers was conducted by assessing structural parameters (Fig.S5). As mentioned in the previous section (S4), the angle ϑ, the distance d and the apparent height were measured (Table S1) for oligomers that are either isolated or close-packed into 2D islands.

Regarding the individual isolated oligomers, the distance d between neighboring units and the apparent heights are similar, independent of whether they were grown by heating or via photo-polymerization. The same is valid for close-packed polymers.

However, a significant change of the bending angle ϑ between isolated and close-packed oligomers is observed. This angle is about a factor 2 smaller in the case of oligomers that are close-packed. Note that the internal oligomer structure is very sensitive to the local environment. When surrounded by other oligomers and individual Br atoms, the single anthracene constituent units are bent closer to the polymer axis with the respect to the case of a single isolated oligomer. This effect of “wire straightening” and oligomer ordering in close-packed arrangements might be ascribed to oligomer-halogen interaction[10].

6. Polymer length distributions (light- and thermally-induced)

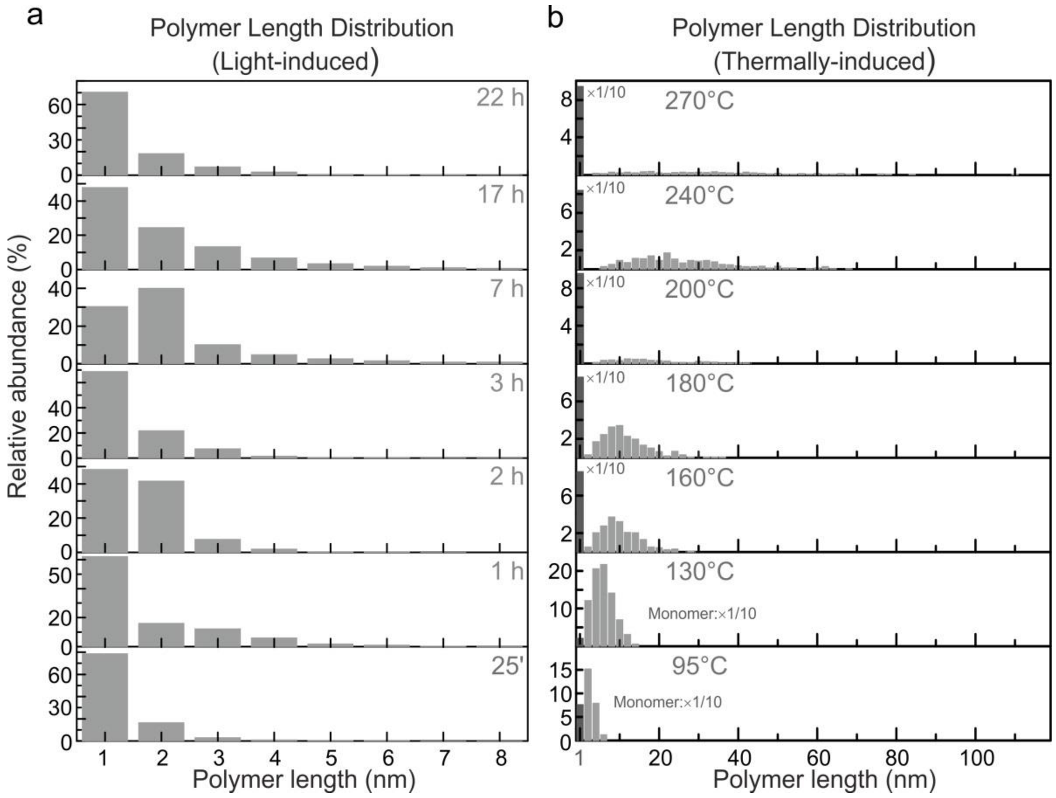

Figure S6. Length histogram distributions of the oligomers obtained by UV laser light illumination (a) or heating (b). The relative abundance of intact monomers is included in both cases. For better clarity, the monomer peak intensity is reduced by a factor 10 in panel (b) and its relative bin indicated in dark gray.

According to the STM measurements, intact monomers (i.e. with both Br substituents attached) are typically the most abundant species on the surface, independent from the external stimulus (light or temperature) used to trigger the debromination of the molecular precursors (Figures S6a,b). On the other hand, XPS measurements reveal the completion of the debromination process at about 200 °C (see Fig.1 in the main text and Fig.S1). This discrepancy is ascribed to a deviation in the sample temperature measurement in the two experiments, which were done in different UHV systems (at the University of Graz and at the ELETTRA synchrotron facility in Trieste, respectively). The Au(111) crystal was mounted on different sample holders when used for STM and XPS measurements. Regarding the XPS measurements, the sample temperature is measured by a thermocouple directly mounted on the sample. In the STM sample holder, the thermocouple is anchored to a clamp holding the sample. This generates a temperature gradient, and the real sample temperature is likely lower than the measured one.

For each experiment conducted by thermal annealing or UV illumination, high-resolution STM images were acquired. The number N of species (monomers and polymers) were counted and the length of each polymer was measured.

Thermal-approach							
T(°C)	25	130	160	180	200	240	270
N	930	355	2207	1620	6975	2037	4001
**Photoapproach**							
Time	25’	1h	2h	3h	7h	17h	22h
N	2862	5013	7447	7493	10527	3819	14047

Table S2. Number N of species (intact molecules and polymers) counted for each experiment as a function of light and temperature.

7. Polymer distribution length (photoapproach)

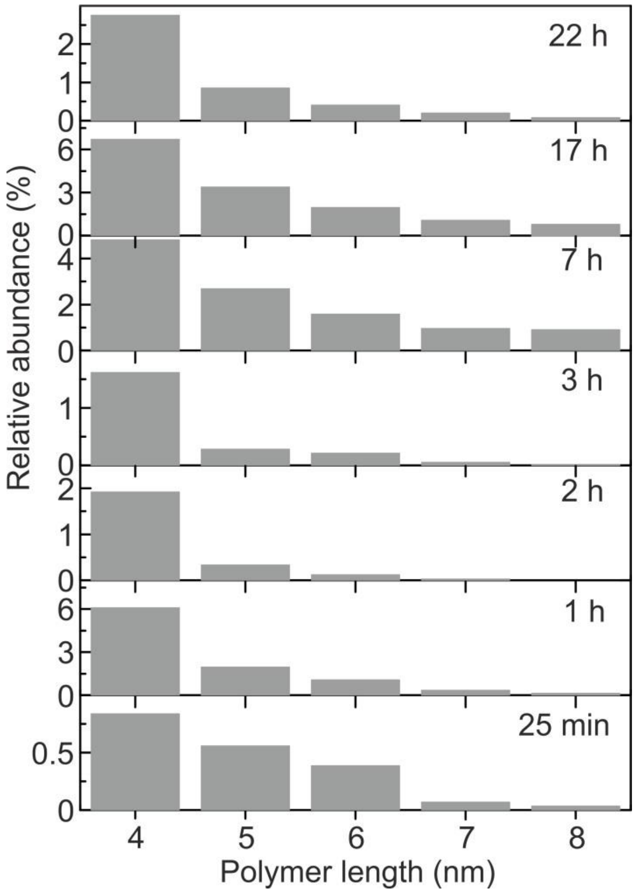

Figure S7. A zoom-in of the polymer length distribution tails of Fig.4a.

A zoom-in of the polymer length distribution tails of Fig.4a is shown in Fig.S7. The distribution is restricted to oligomers with a length between 4 and 8 nanometers. The relative abundances are very small, compared to the oligomers comprised by few anthracene units (Fig.4a), but nevertheless all distributions qualitatively follow the same behavior. Oligomers that are 9-10 nm long (not shown) are found with a relative abundance of only about 0.1%. Longer oligomers (11-13 nm) are found after extended UV illuminations, but with a relative abundance of about 0.01%.

8. Debromination by voltage pulses

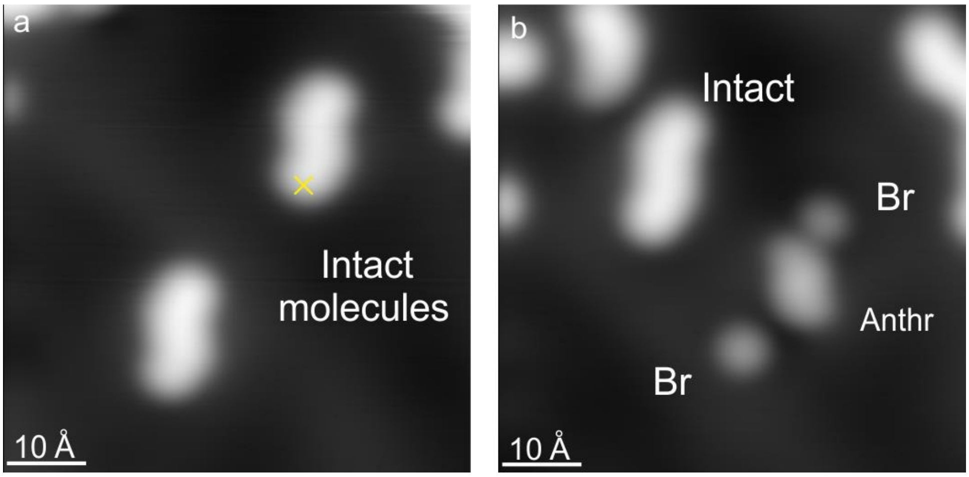

Figure S8. (a) STM image of an area with two isolated and intact DBA molecules. The STM tip was frozen at the cross position and the bias voltage ramped up to 2.5 V. (b) The anthracene and two Br atoms (reaction products) are clearly visible in (b).

Molecule debromination can be induced by positioning the STM tip above the Br position (see yellow cross in Fig.S8a) and ramping the bias voltage up to 2.5 V. After pulsing (Fig.S8b), one of the two molecules looks still intact, but is displaced from its original position while the second one is fully debrominated. In the latter case, the anthracene and the two Br atoms show typically minor lateral displacement from each other.

9. Photo-debromination efficiency at room temperature and 77 K

DBA molecules were deposited onto Au(111) kept at room temperature. Then the sample was cooled down to 77 K and illuminated continuously for 6 h with UV (266 nm) light: afterwards 35.4% of the individual species results (partially or completely) debrominated while the rest is still intact (number of examined molecules = 1965).

The same experiment conducted on Au(111) kept at room temperature during illumination with the same light source (UV; 266 nm) leads to about 35% of the species on the surface being debrominated. Accordingly, the light-induced debromination efficiency is comparable in both cases.

10. Porous networks formation at 77 K

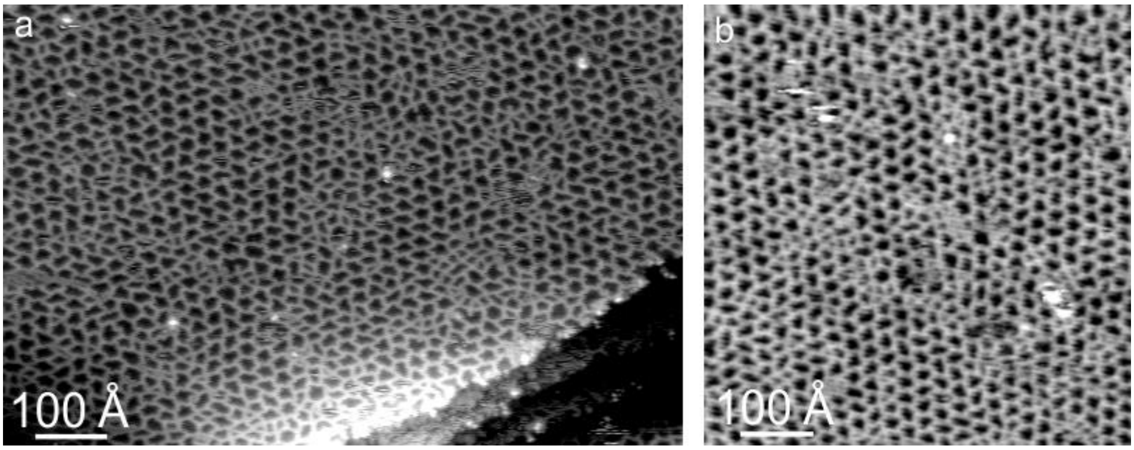

Figure S9. (a,b) STM overview images of DBA molecules on Au(111) taken at 77 K. Molecules were deposited on Au(111) kept at 77 K.

If the molecules are deposited onto the clean Au(111) surface kept at 77 K, porous networks are observed (Fig.S9). Their structure is equivalent to the porous networks observed after deposition at room temperature (see Figures 1b,d in the main text). The formation of these networks at low temperatures shows that even at 77 K, intact DBA molecules still have enough energy to diffuse across the surface and self-assemble into large assemblies.

References

(1) Doyle, C. M.; McGuinness, C.; Lawless, A. P.; Preobrajenski, A. B.; Vinogradov, N. A.; Cafolla, A. A. Surface Mediated Synthesis of 2D Covalent Organic Networks: 1,3,5-Tris(4-Bromophenyl)Benzene on Au(111). *Phys. Status Solidi Basic Res.* **2019**, *256* (2), 1–11.

(2) Smykalla, L.; Shukrynau, P.; Korb, M.; Lang, H.; Hietschold, M. Surface-Confined 2D Polymerization of a Brominated Copper-Tetraphenylporphyrin on Au(111). *Nanoscale* **2015**, *7* (9), 4234–4241.

(3) El-Sayed, A.; Borghetti, P.; Goiri, E.; Rogero, C.; Floreano, L.; Lovat, G.; Mowbray, D. J.; Cabellos, J. L.; Wakayama, Y.; Rubio, A. et al. Understanding Energy-Level Alignment in Donor-Acceptor/Metal Interfaces from Core-Level Shifts. *ACS Nano* **2013**, *7* (8), 6914–6920.

(4) Winkler, S.; Frisch, J.; Schlesinger, R.; Oehzelt, M.; Rieger, R.; Räder, J.; Rabe, J. P.; Müllen, K.; Koch, N. The Impact of Local Work Function Variations on Fermi Level Pinning of Organic Semiconductors. *J. Phys. Chem. C* **2013**, *117* (43), 22285–22289.

(5) Smerieri, M.; Píš, I.; Ferrighi, L.; Nappini, S.; Lusuan, A.; Di Valentin, C.; Vaghi, L.; Papagni, A.; Cattelan, M.; Agnoli, S. et al. Synthesis of Graphene Nanoribbons with a Defined Mixed Edge-Site Sequence by Surface Assisted Polymerization of (1,6)-Dibromopyrene on Ag(110). *Nanoscale* **2016**, *8* (41), 17843–17853.

(6) Li, S.; Czap, G.; Wang, H.; Wang, L.; Chen, S.; Yu, A.; Wu, R.; Ho, W. Bond-Selected Photodissociation of Single Molecules Adsorbed on Metal Surfaces. *Phys. Rev. Lett.* **2019**,

122 (7), 1–6.

(7) Moreno, C.; Panighel, M.; Vilas-Varela, M.; Sauthier, G.; Tenorio, M.; Ceballos, G.; Peña, D.; Mugarza, A. Critical Role of Phenyl Substitution and Catalytic Substrate in the Surface-Assisted Polymerization of Dibromobianthracene Derivatives. *Chem. Mat.* **2019**, *31* (2), 331–341.

(8) Simonov, K. A.; Vinogradov, N. A.; Vinogradov, A. S.; Generalov, A. V.; Zagrebina, E. M.; Mårtensson, N.; Cafolla, A. A.; Carpy, T.; Cunniffe, J. P.; Preobrajenski, A. B. Effect of Substrate Chemistry on the Bottom-up Fabrication of Graphene Nanoribbons: Combined Core-Level Spectroscopy and STM Study. *J. Phys. Chem. C* **2014**, *118* (23), 12532–12540.

(9) Bronner, C.; Björk, J.; Tegeder, P. Tracking and Removing Br during the On-Surface Synthesis of a Graphene Nanoribbon. *J. Phys. Chem. C* **2015**, *119* (1), 486–493.

(10) Abyazisani, M.; MacLeod, J. M.; Lipton-Duffin, J. Cleaning up after the Party: Removing the Byproducts of On-Surface Ullmann Coupling. *ACS Nano* **2019**, *13* (8), 9270–9278.

(11) Tran, B. V.; Pham, T. A.; Grunst, M.; Kivala, M.; Stöhr, M. Surface-Confined [2 + 2] Cycloaddition towards One-Dimensional Polymers Featuring Cyclobutadiene Units. *Nanoscale* **2017**, *9* (46), 18305–18310.

(12) Mairena, A.; Baljozovic, M.; Kawecki, M.; Grenader, K.; Wienke, M.; Martin, K.; Bernard, L.; Avarvari, N.; Terfort, A.; Ernst, K. H. et al. The Fate of Bromine after Temperature-Induced Dehydrogenation of on-Surface Synthesized Bisheptahelicene. *Chem. Sci.* **2019**, *10* (10), 2998–3004.

(13) Batra, A.; Cvetko, D.; Kladnik, G.; Adak, O.; Cardoso, C.; Ferretti, A.; Prezzi, D.; Molinari, E.; Morgante, A.; Venkataraman, L. Probing the Mechanism for Graphene Nanoribbon Formation on Gold Surfaces through X-Ray Spectroscopy. *Chem. Sci.* **2014**, *5* (11), 4419–4423.

(14) Basagni, A.; Sedona, F.; Pignedoli, C. A.; Cattelan, M.; Nicolas, L.; Casarin, M.; Sambi, M. Molecules − Oligomers − Nanowires − Graphene Nanoribbons: A Bottom-Up Stepwise On-Surface Covalent Synthesis Preserving Long-Range Order. *J. Am. Chem. Soc.* **2015**, *137*, 1802–1808.
